# A dataset of textile fibres transferred between garments in a simulated assault for forensic interpretation and statistical analysis

**DOI:** 10.1016/j.dib.2024.110992

**Published:** 2024-10-11

**Authors:** Victoria Lau, Xanthe Spindler, Claude Roux

**Affiliations:** Centre for Forensic Science, University of Technology Sydney, PO Box 123, Ultimo, NSW 2007, Australia

**Keywords:** Textile fibres, Forensic science, Trace evidence, Microtrace, Transfer

## Abstract

The data presented corresponds to the paper “The transfer of fibres between garments in a choreographed assault scenario” [1]. The dataset describes the features (generic fibre type, colour, length, recovered location) of 26101 fibres transferred and recovered following choreographed assault simulations performed by participants wearing a cotton T-shirt and polyester/cotton hoody. This data has been made available so others may employ different statistical methods and modelling approaches to further investigate relationships amongst variables, including continuous length of transferred target and extraneous fibres. Furthermore, forensic practitioners and researchers may use this data for training, teaching and in the evaluation of forensic fibre findings.

Specifications Table

**Table:** 

Subject	Forensic Science; Statistics and probability.
Specific subject area	Textile fibre transfer in simulations modelling common assault*.*
Type of data	Raw numeric data: Table (XLSX file).
Data collection	Choreographed assault simulations were performed by pairs of participants to transfer fibres between a cotton T-shirt and polyester/cotton hoody. Garments were collected immediately after and external surfaces tapelifted to recover fibres for examination. Textile fibres in ten 1 × 1 cm squares per tapelift were visually counted and characterised by generic type and colour. Instruments used were low-powered (Leica EZ4D stereomicroscope, Leica Microsystems GmH, Wetzler) and high-powered microscopes (Leica DM4M-FSCB comparison microscope). Digital images were acquired and processed using instrumental software Leica Application Suite (LAS V4.13.0) with measurement add-on module to measure the length of each fibre, annotated and tabulated*.*
Data source location	Centre for Forensic Science, University of Technology Sydney, Ultimo, Sydney Australia*.*
Data accessibility	Repository name: UTS Research Data Portal Data identification number: 9a7f0f8060ff11efac2c9517142205fd Direct URL to data: https://data.research.uts.edu.au/publication/9a7f0f8060ff11efac2c9517142205fd/558e585e3d2049895029bf8300ccfeab/transfer_raw.xlsx
Related research article	Lau, V., Spindler, X., & Roux, C. (2023). The transfer of fibres between garments in a choreographed assault scenario. Forensic Science International, 349(August), 111,746*.*

## 1 Value of the Data

• The data shows the number, type, length and spatial distribution of textile fibres transferred in a simulated assault under real-world conditions which provides valuable insight into the mechanisms of transfer.
• This data may be used by forensic practitioners and researchers in the calculation of a likelihood ratio (LR) in the evaluation of forensic fibre findings. They may also be used for practitioner training and teaching.
• This is the first known large dataset of length as a continuous variable of transferred target and extraneous fibres. Researchers may use data on this property and of other variables to employ different statistical methods and modelling approaches to further investigate relationships.
• The dataset and novel experimental design can be used to develop and promote future simulated transfer studies.

## 2 Background

Textile fibres are an important physical trace commonly encountered in criminal investigations. Their tendency to readily shed and transfer upon contact affords them value for making associations and reconstructing past events. However, the interpretation of fibre evidence remains a challenge in forensic science. The transfer of fibres has largely been studied by mechanical simulations in controlled environments which do not reflect the uncontrolled nature of criminal events. Furthermore, the subject fibres of such studies may not be contemporaneously relevant in modern society. This creates a need for relevant data to support the interpretation of fibre findings and address questions of activity. Consequently, the objective of the research study was to quantitatively and qualitatively profile the transfer of textile fibres between garments worn by participants simulating a frontal assault scenario*.*

## 3 Data Description

The dataset corresponds to parameters of all fibres examined and characterised. In the repository, data from all experimental replicates are given in the Excel workbook *transfer_raw.xlsx*, with one row per individual fibre.

**Fig. 1:**
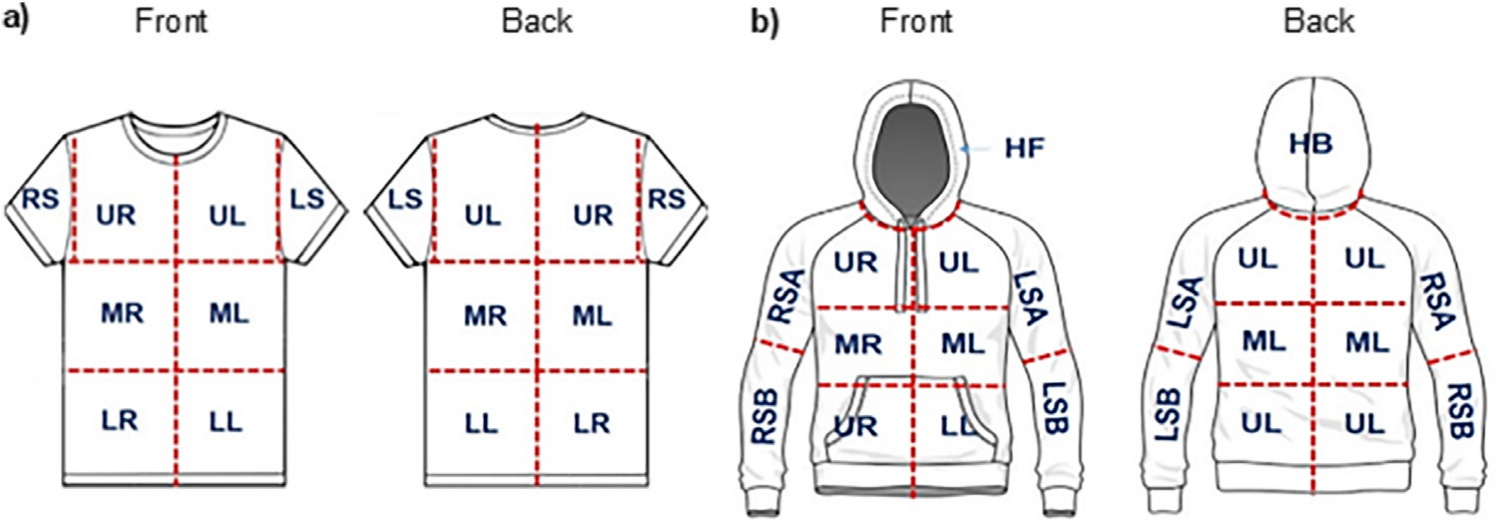
Division of front and back surfaces of worn a) T-shirts into eight (8) zones, and b) hoodies into eleven (11) zones each.

The information provided includes: concatenated experimental code, experimental replicate (A to G), tapelifting zone, front or back of garment surface, lateral location (left, middle, right), axial location (lower, middle, upper), garment size (XS, S, M, L), garment type (T-shirt, hoody) and tapelifting grid (1–10). The proceeding columns present the characteristics of fibre colour, generic type, continuous length and categorical length. Table 1 details the categories used for classification; and Fig. 1 depicts the zonal regions.

**Table 1:** Classification of fibres recovered.

Property	Categories
Fibre colour	Black/grey (bk), blue (bl), dark blue (dbl), turquoise (trq), green (grn), red, purple (pur), orange/brown (org), yellow (ylw), other
Fibre type	Cotton, linen/flax, other vegetable (veg), wool, other animal (animal), man-made, other/miscellaneous
Continuous length (mm)	Numerical value
Categorical length	1 (≤ 1.0 mm). 2 (1.0–3.0 mm), 3 (3.1–5.0 mm), 4 (> 5.0 mm)

## 4 Experimental Design, Materials and Methods

The experimental method for fibre transfer and analysis is detailed in [1]. In brief, a choreographed routine simulating an assault between a ‘victim’ and ‘assailant’ was performed by pairs of participants, each wearing a T-shirt (100 % cotton) or hoody (80 % cotton, 20 % polyester), respectively. The routine was enacted for one minute and garments collected immediately after.

Front and back outer surfaces of garments were divided into zones (Fig. 1), each tapelifted with a single length of adhesive tape using the “press and rub method” to recover fibres.

Each tapelift corresponding to a zone was examined with low-powered microscopy (80 – 320x). Fibres in ten randomly selected 1 × 1 cm squares along the length of each tape were marked, counted and classified according to colour and generic type (Table 1). Digital micrographs were acquired of each grid and marked fibres. Fibre classification was further verified by mounting fibres in glycerol on glass slides for examination under high-powered microscopy (50–1000×).

Continuous length of each individual fibre was measured using the manual measurement software module (LAS V14.3.0). This module enables the user to manually trace an object on a captured image and provide a live measurement to be annotated on the image and saved as tabulated data. Recorded lengths were tabulated using Microsoft Excel and categorised into four size ranges of less than or equal to 1.0 mm, 1.0–3.0 mm, 3.0–5.0 mm and greater than or equal to 5.0mm.

## Limitations

The time- and labour-intensive nature of fibre analysis has limited the compilation of a complete dataset, whereby small and/or damaged fibres were unable to be identified by generic type. As characterisation was achieved by visual examination of morphological features, more extensive instrumental physical and chemical analysis is required for comprehensive classification of natural- and man-made fibre subtype.

Fibres were counted in ten randomised centimetre squares of each tapelift, and thus the data is indicative of the quantity of fibres implicated in transfer, and not to be interpreted as an accurate measure. This limitation presents potential for further work accounting for all fibres recovered.

## Ethics Statement

All human subjects were briefed to the research objectives and provided informed consent to participate in the research. All experiments were approved by the University of Technology Sydney Human Research Ethics Committee (HREC ETH18–3059).

## CRediT authorship contribution statement

**Victoria Lau:** Methodology, Formal analysis, Investigation, Writing – original draft, Writing – review & editing, Visualization. **Xanthe Spindler:** Conceptualization, Supervision, Writing – review & editing, Project administration. **Claude Roux:** Conceptualization, Supervision, Writing – review & editing, Project administration.

## Data Availability

UTS Research Data PortalFIbre Transfer Data (Original data). UTS Research Data PortalFIbre Transfer Data (Original data).

## References

[1] Lau V., Spindler X., Roux C. (2023). The transfer of fibres between garments in a choreographed assault scenario. Forensic Sci. Int..

